# CAREGIVING BURDENS OF TASK TIME AND TASK DIFFICULTY AMONG PAID AND UNPAID CAREGIVERS OF PERSONS LIVING WITH DEMENTIA

**DOI:** 10.1093/geroni/igac059.3121

**Published:** 2022-12-20

**Authors:** Matthew Smith, Chung Lin Kew, Gang Han, Lucas Wilson, Shinduk Lee, John Fitch, Marcia Ory

**Affiliations:** Texas A&M University, College Station, Texas, United States; Texas A&M University, College Station, Texas, United States; Texas A&M University, College Station, Texas, United States; Texas A&M University, College Station, Texas, United States; University of Utah, Salt Lake City, Utah, United States; Texas A&M University, College Station, Texas, United States; Texas A&M University, College Station, Texas, United States

## Abstract

Demands of caregivers of persons living with dementia (PLWD) are often influenced by the context of their caregiving situation. This study examines factors associated with caregiving burden in terms of task time and task difficulty among paid and unpaid caregivers of PLWD. Cross-sectional baseline survey data were analyzed from 110 paid and unpaid caregivers of PLWD participating in a larger NIH-funded study assessing the feasibility of using a novel in-situ sensor system. Oberst Caregiving Burden Scale constructs of task time and task difficulty served as dependent variables. Two least squares regression models were fitted, controlling for contextual items related to the caregiver, care recipient, and caregiving logistics. Caregivers whose care recipients were female (B=-0.29, P=0.006), had more chronic conditions (B=0.31, P=0.011), and had lower Mini-Mental State Exam scores (B=-0.20, P=0.015) reported higher task time burdens. Caregivers whose care recipients had other paid caregivers (B=0.30, P=0.031) and spent more months/years caring for their care recipients (B=0.28, P=0.004) reported higher task time burdens. Caregivers’ task time burden was positively associated with their emotional stress level (B=0.30, P=0.020). Caregivers’ task difficulty burden was positively associated with their emotional stress (B=0.30, P=0.029) and depressive symptomatology (B=0.32, P=0.002). Results reinforce the relationship between caregiver burden and mental health. While the care recipient’s disease profile and needs were drivers of task time burden, which may also require coordination with other paid caregivers, task difficulty was emotionally driven. Findings highlight the importance of caregiver support services and programming for mental health.

